# Hybrid [^18^F]FDG PET/MR Imaging Parameters for the Prediction of Tissue Biomarkers in Invasive Ductal Breast Cancer

**DOI:** 10.3390/bioengineering13040435

**Published:** 2026-04-08

**Authors:** Ilaria Neri, Francesca Gallivanone, Elena Venturini, Carla Canevari, Chiara Caleri, Nicole Rotmensz, Samuele Ghezzo, Carolina Bezzi, Paola Mapelli, Pietro Panizza, Maria Picchio, Rosa Di Micco, Arturo Chiti, Oreste Davide Gentilini, Paola Scifo

**Affiliations:** 1 Nuclear Medicine Department, IRCCS San Raffaele Scientific Institute, 20132 Milan, Italybezzi.carolina@hsr.it (C.B.);; 2 Institute of Bioimaging and Complex Biological Systems, National Research Council (IBSBC-CNR), 20054 Segrate, Milan, Italy; 3 Breast Radiology Unit, IRCCS San Raffaele Scientific Institute, 20132 Milan, Italy; 4 Breast Surgery Unit, IRCCS San Raffaele Scientific Institute, 20132 Milan, Italy; 5 Faculty of Medicine and Surgery, Vita-Salute San Raffaele University, 20132 Milan, Italy

**Keywords:** hybrid PET/MRI parameters, breast cancer, invasive ductal carcinoma, breast cancer biomarkers

## Abstract

Breast cancer (BC) requires the evaluation of tumor aggressiveness features to guide treatment decisions. Biopsy-derived prognostic information may differ from surgical histopathology due to tumor heterogeneity. Hybrid PET/MRI can provide additional information for tumor characterization, supporting initial therapy planning and prognosis. In this work, we acquired 157 BC patients using a hybrid PET/MRI scanner. The PET data were combined with ADC and semi-quantitative DCE-MRI metrics to derive “hybrid PET/MRI parameters.” Pathological data such as tumor grade, hormone receptors, proliferation index (Ki67), and surrogate molecular subtype were collected, and we evaluated their associations with hybrid imaging, also comparing with the PET and MRI data analyzed separately. Ki67 showed moderate correlations with PET, ADCmin, and most hybrid parameters. The PET and hybrid data differentiate histopathological factors, while ADCmin differentiates G1 vs. G2 and luminal A vs. luminal B. In the ROC analysis, hybrid SUVmax/ADCmin shows better performance to predict luminal B from luminal A (AUC 0.720, sensitivity 73.1%, specificity 63.2%, PPV 54.3%, NPV 79.7%) than SUVmean alone. Our findings suggest that these novel hybrid PET/MRI parameters may help the characterization of tumor tissue in IDC. However, a multivariate analysis is needed to confirm our preliminary results.

## 1. Introduction

Breast cancer (BC) is the most diagnosed cancer in women worldwide, accounting for 2.3 million new cases and more than 650 thousand cases of death in 2020 [1]. To choose the appropriate treatment for biopsy-proven BC patients, a series of features of aggressiveness extracted from the tumor are identified from histology, such as tumor grade, estrogen receptor (ER), progesterone receptor (PgR), human epidermal growth factor receptor 2 (HER2+) status, and proliferation index (Ki67) [2]. These factors help to improve the characterization of tumor heterogeneity, and they can be used as prognostic markers for recurrence risk, contributing to the decision of the therapy to follow. Surgery remains the initial treatment for most women diagnosed with early-stage tumor [2]. However, despite core needle biopsy being the best procedure for BC diagnosis [3], prognostic information obtained from it may be discordant with that extracted from surgery due to the small sample size and tumor heterogeneity [4,5].

Hybrid positron emission tomography/magnetic resonance imaging (PET/MRI) acquires PET and MR images simultaneously, providing functional and morphological information in a single clinical session with less radiation compared to PET/computer tomography (CT). In breast imaging, PET with [^18^F]FDG is used for staging and restaging purposes, evaluation of tumor extent, and treatment response in metastatic disease [6,7,8,9]. Typical semi-quantitative measures of glucose uptake in a specific region are the maximum and mean standardized uptake values (SUVmax and SUVmean), in addition to Metabolic tumor volume (MTV) and total lesion glycolysis (TLG) [10]. Nevertheless, lesion size may influence the sensitivity for the initial BC diagnosis due to the limited spatial resolution of PET imaging (partial volume effect) that may decrease in lesions of small size [8,9]. On the other hand, MR is typically used in breast imaging for the diagnosis and evaluation of tumor response to treatment [11]. Thanks to multiparametric MRI, different qualitative and quantitative information can be obtained from the examination. Dynamic contrast-enhanced (DCE)-MRI and diffusion-weighted imaging (DWI) are routinely included in breast protocols. The former uses gadolinium uptake inside the tumor to extract semi-quantitative or quantitative parameters. DCE-MRI is very sensitive to breast lesion detection, but has moderate specificity [12]. DWI provides information on water molecule diffusion inside tissues that can be quantitatively measured through apparent diffusion coefficient (ADC) maps. A restriction of diffusivity is typically shown in malignant BC, where a low ADC value is observed [13,14]. Hence, the information provided by both [^18^F]FDG PET and MRI could improve tumor characterization and may lead to outcome prediction [9].

Rather than replacing biopsy, imaging can provide additional information for tumor assessment, contributing to the initial therapy planning and prognosis [6]. Several PET/MRI studies [15,16,17,18,19,20,21,22,23,24] have evaluated BC phenotype with imaging parameters extracted from PET and MR images, exploring their associations with prognostic factors. Among these studies, two [21,22] investigated the so-called “hybrid parameters”, which integrate in a single value both PET and MRI quantitative information. However, in these two works [21,22], PET and MR imaging parameters were obtained from two separated scanners ([^18^F]FDG PET/CT and MR). One BC study [25] investigated the role of “hybrid parameters” extracted from a simultaneous PET/MRI scanner but focusing on the prediction of treatment response rather than to evaluate their associations with prognostic factors. Furthermore, PET data were combined with quantitative perfusion DCE-MRI [21] and ADC-only parameters [22,25].

In the present study, we used “hybrid parameters” obtained from PET and MR images acquired by a simultaneous PET/MR scanner to investigate their relationships with histopathological factors. Moreover, we combined the PET data with both ADC and semi-quantitative parameters derived from the time–intensity curve (TIC) of DCE-MRI.

Therefore, the aim of the study was to investigate whether BC tissue biomarkers are associated with PET, MRI, and combined PET/MR imaging parameters in a group of patients with invasive ductal carcinoma (IDC) and if hybrid imaging biomarkers predict them better than PET and MR data taken separately.

## 2. Materials and Methods

### 2.1. Patients

The San Raffaele Ethical Committee has approved the study on 7 March 2019, and all patients provided written informed consent to participate in the study. Female patients with biopsy-proven early BC were enrolled from July 2020 to May 2024 in a prospective clinical trial (ClinicalTrials.gov Identifiers: NCT04829643) at San Raffaele Hospital in Milan [26,27]. The recruited patients with diagnosis of early invasive breast cancer were candidate to upfront surgery for comparing PET/MRI and sentinel node biopsy in staging the axilla. The inclusion and exclusion criteria of this clinical trial are described here [27]. Our analysis is an unplanned study of the tertiary endpoint of the clinical trial [27], and it was conducted on a homogeneous dataset of patients affected by IDC. Since one of the aims of this work is to compare the performance of different combinations of imaging parameters to predict tissue biomarkers, we included only lesions that have complete imaging and histopathological data.

### 2.2. [^18^F]FDG PET/MRI Acquisition

The PET/MRI acquisition was performed 1–14 days prior to surgery. All patients fasted 6 h before the acquisition started. [^18^F]FDG (3.7 MBq/kg body weight) was injected 60 min before the scanning, and blood glucose levels were measured. Then, patients underwent [^18^F]FDG PET/MRI using a fully hybrid PET/MRI 3T system (SIGNA PET/MR, GE Healthcare, Waukesha, WI, USA). The acquisition protocol consisted in: (1) whole body exam with the patient in supine position, with arms along the body, using a combination of coils, followed by (2) prone acquisition of high statistics breast PET scan with diagnostic breast MR examination, using a dedicated eight-channel breast MR coil. The total amount of time for the entire PET/MRI acquisition was about 1 h and 10 min.

The total body (TB) PET in the supine protocol consists of the acquisition of five or six FOVs (each PET bed lasts 4 min/FOV) to identify lesions in the whole body. For each bed, TB T1-weighted LAVA-Flex sequence and TB DWI with b = 50–900 s/mm^2^ were acquired simultaneously to PET.

In the prone protocol, PET acquisition lasts 20 min. During PET scan, the following MR pulse sequences were acquired:

- Axial T2-weighted FSE (fast spin echo) sequence: TR = 9924 ms, TE = 138.4 ms, matrix size = 384 × 384, FOV = 36.1 × 36.1 cm^2^, slice thickness = 3.3 mm;
- DWI-EPI (echo planar imaging): TR = 5251 ms, TE = 79.4 ms, matrix size = 96 × 96, FOV = 36 × 36 cm^2^, slice thickness = 4.3 mm, b-values = 0–1000 s/mm^2^, and parallel imaging factor = 2.
- DCE imaging with contrast agent injection (gadolinium-DO3A-butriol, Gadovist© 1.0, Schering AG, Berlin, Germany). Each dynamic set included one pre-contrast and, after gadolinium injection, five post-contrast images. DCE-MR images were acquired with VIBRANT-Flex sequence: TR = 8.1 ms, TE = 1.8 ms, matrix size = 352 × 352, FOV = 36 × 36 cm^2^, slice thickness = 2 mm, time resolution = 90 s.

PET images were reconstructed using Bayesian penalized likelihood PET reconstruction algorithm (Q.Clear, β = 250), matrix = 256 with TOF implementation. Attenuation correction was performed using the MR-based LAVA-Flex sequence.

### 2.3. PET/MR Image Processing and Tumor Segmentation

Two imaging experts (Ca.C. and E.V.), a nuclear medicine physician, and a radiologist segmented breast lesions on PET and MR images, respectively, using 3DSlicer [28].

Specifically, PET images were semi-automatically segmented for the definition of a 3D lesion volume (volume of interest, VOI) using a fixed-threshold methodology with a cutoff of 40% from the maximum uptake value, following manual adjustment [29,30]. SUVmean and SUVmax were normalized by body weight and calculated for each VOI.

For MRI, first, DCE-MRI post-contrast images were subtracted to the pre-contrast one to better visualize BC. Then, breast lesions were manually segmented on the subtracted images generated between the second post-contrast and pre-contrast phases. Finally, the VOIs were cloned on the six DCE-MR phases and manually adjusted. In patients where motion occurred due to voluntary or involuntary movements, the segmentation did not match with the lesion in all or some phases. Therefore, a different VOI was drawn in each phase.

ADC maps were generated from the DW images acquired using the manufacturer-supplied software on the PET/MRI console. Lesions were manually segmented on the DWI b = 1000 s/mm^2^ and then cloned on the ADC map. Due to eddy current effects [31], diffusion images corresponding to different b-values were misaligned for 12 lesions. Therefore, for these patients, image registration was performed using the 3DSlicer software (vers.4.11, rev.29738) [28] to co-register the lesion on DWI b = 0 s/mm^2^ and on DWI b = 1000 s/mm^2^. Specifically, rigid transformations were applied on the DWI b = 1000 s/mm^2^ image and its lesion segmentation to match the lesion on DWI b = 0 s/mm^2^. The nearest neighbor interpolator [32] was used. Finally, the corresponding ADC map was generated. Figure S1 in Supplementary Material shows an exemplar case.

### 2.4. Extraction of Imaging Parameters

For each lesion, two PET parameters (SUVmax and SUVmean) and two ADC parameters (ADCmin and ADCmean) were calculated. Semi-quantitative analysis was performed on DCE-MRI series and the following five DCE parameters were calculated for each VOI:

- First post-contrast SI enhancement percentage (E_1_) [33]:
  (1) $$E_{1}=\frac{100\times {\;SI}_{1}-{SI}_{0}}{{SI}_{0}},$$
  where SI_0_ is the mean SI inside VOI in the pre-contrast phase, and SI_1_ is the mean SI inside VOI in the first post-contrast phase.
- Early signal enhancement ratio (ESER) [33]:
  (2) $$ESER=\frac{100\times {SI}_{1}-{SI}_{0}}{{SI}_{2}-{SI}_{0}},$$
  where SI_2_ is the mean SI inside VOI in the second post-contrast phase.
- Slope [33]:
  (3) $$Slope=\frac{100\times {SI}_{5}-mean({SI}_{1},{SI}_{2})}{mean({SI}_{1},{SI}_{2})},$$
  where SI_5_ is the mean SI inside VOI in the fifth post-contrast phase.
- Signal peak enhancement ratio (SPER) [33]:
  (4) $$SPER=\frac{{SI}_{peak}-{SI}_{0}}{{SI}_{5}-{SI}_{0}},$$
  where SI_peak_ is the mean SI inside VOI of the peak (maximum) enhancement between the six phases.
- Signal first enhancement ratio (SFER) [34]:
  (5) $$SFER=\frac{{SI}_{1}-{SI}_{0}}{{SI}_{5}-{SI}_{0}}.$$

For the analysis, we combined the PET and MRI data to explore the synergy of the information provided by using a hybrid PET/MRI. The mathematical formulations of the hybrid parameters are dependent on the behaviors of PET and MR imaging in malignant breast tumors, reflecting their biology. Specifically, malignant breast lesions are generally characterized by increased PET SUV [35] and a low ADC [36]. Moreover, the TIC of a typical malignant breast lesion presents a fast wash-in phase immediately after contrast agent injection, followed by a reduced or stable signal intensity (“wash-out” or “plateau”, respectively [37,38]). Given this trend of the TIC and the definitions of the parameters in malignant tumors, the values of E_1_, ESER, SPER, and SFER increase, as they are all indices related to the enhancement after the contrast bolus. Conversely, Slope tends to decrease in most cases. Therefore, to emphasize the “malignancy” of the tumor, we multiplied the SUV by the parameters that increase (E_1_, ESER, SPER, and SFER) and divided the SUV by those that decrease (ADCmin, ADCmean, and Slope). These considerations are based on BIRADS (Breast Imaging Reporting and Data System) and on the results found in the literature [35,36,37,38,39]. Twenty-three imaging biomarkers (PET, MRI, and hybrid PET/MRI) were obtained to perform univariate analyses:

- PET parameters: SUVmax and SUVmean;
- DCE-MRI parameters: E_1_, ESER, Slope, SPER, and SFER;
- ADC parameters: ADCmin and ADCmean;
- Hybrid PET/MRI parameters: SUVmax × E_1_, SUVmean × E_1_, SUVmax × ESER, SUVmean × ESER, SUVmax/Slope, SUVmean/Slope, SUVmax × SPER, SUVmean × SPER, SUVmax × SFER, SUVmean × SFER, SUVmax/ADCmin, SUVmean/ADCmin, SUVmax/ADCmean, and SUVmean/ADCmean.

### 2.5. Pathological Analysis

For each tumor, the histotype was assessed and the following biomarkers were assessed: tumor grade, ER, PgR, Ki67, HER2+ status, and the surrogate molecular subtype. ER, PgR, and Ki67 percentage values are represented as mean value ± standard deviation (sd). Both ER and PgR are defined as negative if their value is equal to 0%, otherwise, they are defined as positive. The Ki67 value was dichotomized as positive if Ki67 ≥ 20%, otherwise, it was defined as negative. The HER2+ status is considered negative if its score is 0 or 1+, and it is considered positive if the score is 3+. Where HER2+ equals 2+, the fluorescence in situ hybridization analysis was performed to detect the amplification status. Regarding the analysis on the surrogate molecular subtype, only luminal A and luminal B were investigated due to their numerical prevalence compared to the other surrogate molecular subtypes, and they were defined as follow:

- Luminal A: ER positive and/or PgR positive, Her2 negative and low Ki67 (<20%);
- Luminal B: ER positive and/or PgR positive, Her2 negative and high Ki67 (≥20%).

### 2.6. Statistical Analyses

Statistical analyses were performed with Python (version 3.9). Kolmogorov–Smirnov test was used to assess normal distribution of variables. Spearman’s coefficients (r) were calculated to compare “single-modality” (PET and MRI) with “hybrid” (PET/MRI) imaging parameters and to evaluate the correlation between all imaging biomarkers (PET, MRI, and hybrid PET/MRI) and percentage values of ER, PgR, and Ki67 to find which imaging parameter correlates better with the compared histological factor. Finally, based on distribution of variables, the Mann–Whitney U test was applied to assess the relationship between the imaging parameters and grade (G1 vs. G2, G1 vs. G3, and G2 vs. G3), Ki67 (positive vs. negative), and molecular subtype (luminal A vs. luminal B). In addition to *p*-value for the Mann–Whitney U test, the confidence interval (CI) was calculated using the CI for the Hodges-Lehmann estimate. To correct for family-wise error rate, the Bonferroni method was used for multiple comparisons, and adjusted *p* < 0.05 was considered significant. In the Spearman correlation between the “single” and “hybrid” imaging parameters, we aimed to evaluate the correlation between all imaging parameters. Therefore, in this case, the Bonferroni correction was performed with a factor of 9 × 14 = 126, corresponding to the number of comparisons between “single” (n = 9) and “hybrid” (m = 14) parameters. In the Spearman correlation and Mann–Whitney U Test between histopathological factors and imaging parameters, we aimed to evaluate the associations of each histopathological factor with all imaging parameters (correlations in the case of Spearman analysis and differentiations for Mann–Whitney U Test). Therefore, in these cases, the Bonferroni corrections were performed with a factor of 9 + 14 = 23, corresponding to the total number of image parameters (“single” n = 9 + “hybrid” m = 14). Also, the 95% CI for the Hodges-Lehmann estimate was corrected for the 23 parameters. Therefore, a 99.8% CI was calculated.

After assessing the statistically significant parameters, the receiver operation curve (ROC) analysis was performed, and the area under curve (AUC) was calculated with the corresponding CI. The best cutoff was determined using the Youden’s index.

## 3. Results

### 3.1. Patients

**Figure 1:**
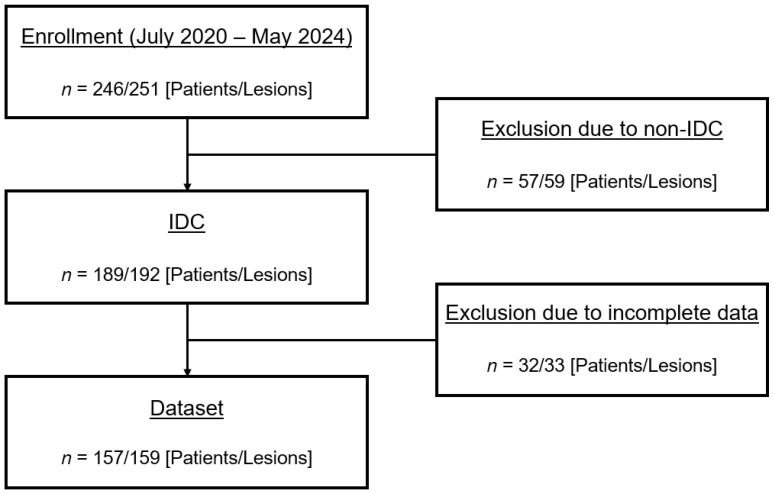
Flowchart of selected patients for this study. IDC: invasive ductal carcinoma.

A total of 246 patients were enrolled from July 2020 to May 2024 [27]. Among this cohort, 189 out of 246 patients had IDC, accounting for a total of 192 breast lesions (three patients presented bilateral tumors). Thirty-three lesions were excluded from the analysis: 26 cases had unsatisfied fat suppression on the DW images due to inadequate shimming [40]; in six cases, the lesions were not visible in DCE or in PET; and in one case, the lesion had incomplete histopathology. Eventually, 157 patients (mean age: 55 ± 11 years) were included in the analysis for a total of 159 IDC breast lesions. The selection procedure of the patients in this study is shown in Figure 1.

### 3.2. Tissue Biomarkers

The mean (±sd) of hormone receptors and proliferation percentage values are: ER 83(±22.4)%, PgR 60(±33.9)%, and Ki67 21(±14.3)%. Fifty-two tumors were classified with grade 1 (G1), 76 with grade 2 (G2), and 31 with grade 3 (G3). Dichotomized immunopathological findings showed 156 lesions with positive ER (ER > 0%), 157 with positive PgR (PgR > 0%), 71 Ki67-positive lesions (Ki67 ≥ 20%), and 14 HER2 status-positive lesions. Finally, the following surrogate molecular subtype were found: 87 luminal A, 52 luminal B, 13 luminal B Her2+, six triple negative, and one Her2+. The number of lesions for each tissue biomarker is shown in Table 1.

### 3.3. Correlation Analysis

Regarding the correlation among imaging parameters, most hybrid PET/MRI parameters exhibited high correlation with PET parameters and low-to-fair correlation with MRI parameters. The complete correlation matrix is available in Table S1 of Supplementary Material.

**Figure 2:**
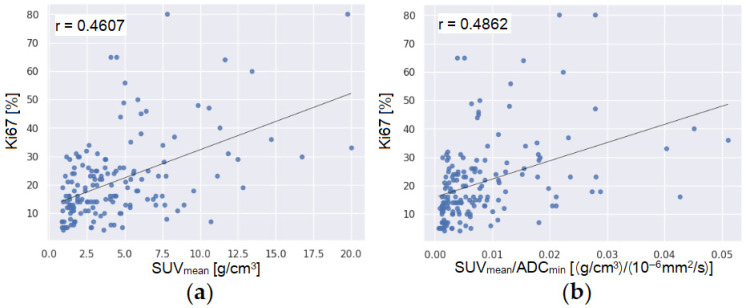
Scatterplots of correlations (**a**) between Ki67 and SUVmean and (**b**) between Ki67 and SUVmean/ADCmin. Their Spearman’s correlation coefficient (r) is shown.

Concerning the correlation between imaging and tissue biomarkers, the percentage value of Ki67 correlated with the PET, MRI, and hybrid PET/MR parameters. PET imaging biomarkers significantly correlated with the Ki67 percentage values. Specifically, both SUVmax and SUVmean showed fair correlation (SUVmax: r = 0.4505, *p* < 0.0001; SUVmean: r = 0.4607, *p* < 0.0001). Among MRI parameters, only ADCmin was significantly correlated with Ki67 (r = −0.2833, *p* < 0.05). Most hybrid PET/MR parameters, with the exception of SUVmax/Slope and SUVmean/Slope, fairly correlated with Ki67 (r ≥ 0.3848, *p* < 0.0001). The strongest correlations were observed for SUVmax/ADCmin (r = 0.4847) and SUVmean/ADCmin (r = 0.4862). Figure 2 shows scatterplots of the highest correlation of Ki67 obtained with “single” (SUVmean, Figure 2a) and “hybrid” imaging parameters (SUVmax/ADCmin, Figure 2b). Finally, after multiple test corrections, no correlations were found between imaging parameters and ER and PgR. A comprehensive summary of the correlation coefficients between imaging parameters and ER, PgR and Ki67 is shown in Table S2 of Supplementary Material.

### 3.4. Tumor Grade, Proliferation Index, and Molecular Subtype Differentiation Analysis

**Figure 3:**
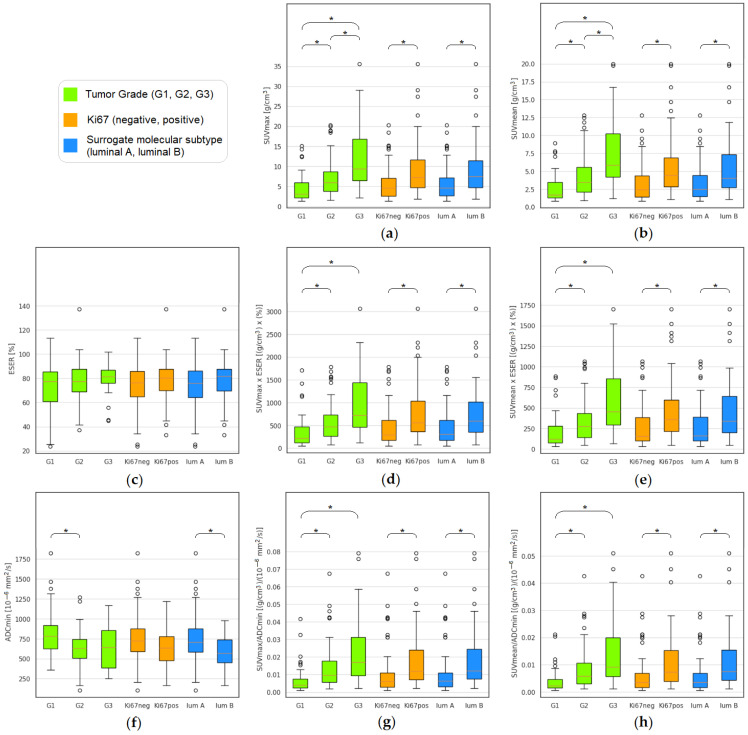
Boxplots showing the distribution of (**a**) SUVmax, (**b**) SUVmean, (**c**) ESER, (**d**) SUVmax × ESER, (**e**) SUVmean × ESER, (**f**) ADCmin, (**g**) SUVmax/ADCmin, and (**h**) SUVmean/ADCmin for each histopathological factor. Significant difference is showed with the “*”.

Univariate analyses were performed for grade, Ki67, and surrogate molecular subtype (luminal A vs. luminal B) differentiation. All adjusted *p*-values are listed in Table 2. Figure 3 displays a series of boxplots showing the distribution of some hybrid imaging parameters (together with the corresponding single PET and MRI data) that reported significant results in the differentiation analysis for each histopathological factor.

Among the single-modality parameters, SUVmax and SUVmean differentiated all histopathological parameters (Figure 3a,b). ADCmin significantly differentiated G1 vs. G2 (*p* = 0.0021, CI: 41–300) and luminal A vs. luminal B (*p* = 0.0235, CI: 8–269) (Figure 3f), while the DCE-MRI parameters are not significant (*p* > 0.05). See ESER boxplots as an example in Figure 3c.

All hybrid imaging biomarkers, except SUVmax/Slope and SUVmean/Slope, differentiated G1 vs. G2, G1 vs. G3, Ki67 positive vs. negative, and the surrogate molecular subtypes (luminal A vs. luminal B) (Figure 3d,e,g,h).

### 3.5. Prediction of Tumor Grade, Ki67, and Surrogate Molecular Subtype

**Figure 4:**
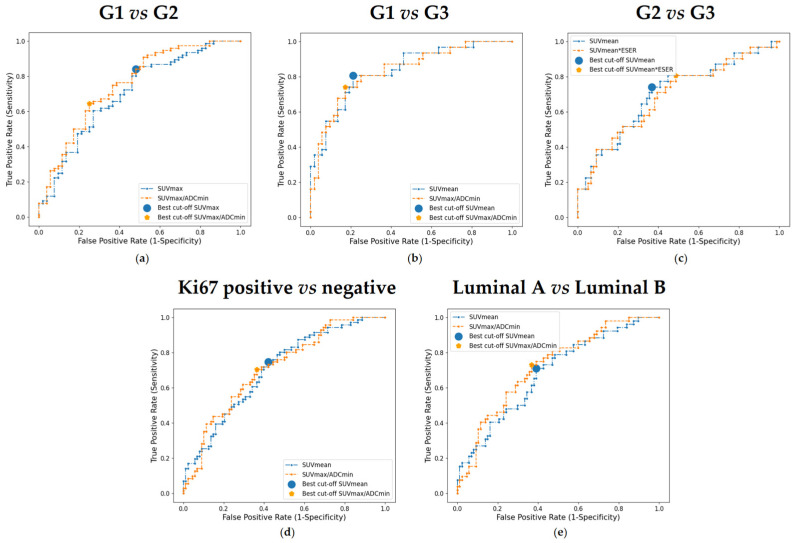
ROC analysis to predict tumor grade, Ki67, and tumor subtype: (**a**) G1 vs. G2; (**b**) G1 vs. G3; (**c**) G2 vs. G3; (**d**) Ki67: positive vs. negative; (**e**) luminal A vs. luminal B. For each plot, the ROC of “single-modality” imaging parameter is depicted in blue, while the curve of “hybrid” imaging parameters is in orange. The optimal cutoff for each curve chosen by the Youden’s index is shown.

To compare the prediction ability between single-modality and hybrid imaging parameters, we calculated the AUC for all imaging biomarkers. For each prediction (G1 vs. G2, G1 vs. G3, G2 vs. G3, Ki67: positive vs. negative, and luminal A vs. luminal B), two parameters with the highest AUC were compared—one for each group (i.e., “single-modality” vs. “hybrid”). The AUC score, together with sensitivity, specificity, positive predictive value (PPV), and negative predictive value (NPV) of the chosen imaging parameters are listed in Table 3, while the ROC analysis performance is shown in Figure 4.

With regard to the tumor grade prediction, SUVmax/ADCmin had an AUC of 0.750 in distinguishing between G1 and G2, and it showed higher specificity and PPV than SUVmax (Figure 4a). On the other hand, in the prediction of G3 from G1, SUVmean and SUVmax/ADCmin had similar AUC (0.836 and 0.833, respectively) but different metric values. Specifically, SUVmean had higher sensitivity and NPV than the hybrid parameter (Figure 4b). Finally, SUVmean showed a slightly increased AUC (0.702) in distinguishing G2 vs. G3 compared to SUVmean × ESER (0.689), with higher specificity and PPV (Figure 4c).

Concerning the prediction of Ki67, both “single-modality” (SUVmean) and “hybrid” (SUVmax/ADCmin) parameters have similar AUC (0.703 and 0.710, respectively). Specifically, SUVmean had higher sensitivity and NPV than SUVmax/ADCmin (Figure 4d).

Finally, considering molecular subtype prediction (luminal A vs. luminal B), SUVmax/ADCmin had higher AUC than SUVmean (0.720 and 0.685, respectively). At their best cutoff points, SUVmax/ADCmin had all metric values higher than SUVmean (Figure 4e).

## 4. Discussion

This study demonstrated the association between PET/MRI biomarkers and the Ki67, tumor grades and surrogate molecular subtypes (luminal A and luminal B) in a homogenous BC group of patients with invasive ductal carcinoma. In addition, we found that hybrid SUVmax/ADCmin imaging biomarker shows higher metric values in discriminating luminal A from luminal B surrogate molecular subtype than SUVmax parameter alone. It is important to underline that, since the present study represents an exploratory and unplanned analysis of a subgroup of breast lesions (IDC) derived from the tertiary endpoint of a trial, the power analysis originally performed for the clinical trial is not specifically calculated for this subgroup. However, we deliberately focused on a subgroup of a specific histotype (namely the most prevalent one [27]) rather than the entire cohort to ensure the analysis of a homogeneous dataset to minimize confounds related to the biology of the tumor.

The idea of combining PET and MRI parameters has been previously investigated in BC studies [21,22,25,41]. Baba et al. [41] combined SUVmax with ADCmean (SUVmax/ADCmean) and found that the hybrid biomarker showed better results in differentiating benign from malignant breast lesions with respect to the two individual parameters alone. Wang et al. [25], who investigated a group of BC patients before and after neoadjuvant chemotherapy cycles, found that the ratios of Δ%TLG (percentage change in TLG)/Δ%ADCmin and Δ%SUVmax/Δ%ADCmin had an AUC score higher than single imaging biomarkers in predicting therapy response. Two BC studies investigated the associations between imaging parameters and prognostic factors [21,22]. Nakajo et al. [22] found that SUVmax/ADCmean could predict a worse prognosis, producing fewer false positive and false negative results compared to SUVmax and ADCmean, despite the fact that the hybrid parameter did not significantly improve the accuracy. An et al. [21] combined PET with DCE-MRI pharmacokinetic parameters, finding that SUVmax/Ve (SUVmax/extravascular extracellular space volume) was the only imaging parameter to differentiate the histologic grade. In contrast to previous studies [21,22,41], which relied on imaging data acquired using separate scanners, our study used imaging data simultaneously acquired by a PET/MRI scanner. The use of a hybrid system allows spatial and temporal registration of the body districts so that multiple imaging biomarkers, in the same pathological condition, can be extracted.

In this study, we focused on a specific BC histotype to have a homogeneous sample. Actually, for all the analyses we have decided to select only IDC patients. As a matter of fact, it is widely asserted that [^18^F]FDG uptake for ILC, tubular carcinoma, and DCIS is lower than in IDC [42]. Moreover, in DCE-MRI, the trend of contrast enhancement in DCIS is variable, and it overlaps with the TIC of benign lesions, with a slow initial phase and a delayed phase, more persistent than IDC [43]. Finally, Woodhams et al. [44] found that the ADC of mucinous carcinomas is significantly higher than ADC values of benign and other malignant tumors. For all these reasons, we decided to include only IDC patients, to avoid biases due to the different behavior of imaging biomarkers dealing with heterogeneous datasets.

In general, our findings are consistent with the previously reported literature. Among the single-modality parameters, we found that ADCmin, SUVmax, and SUVmean are significant in our analyses. Specifically, ADCmin showed a correlation with the percentage value of Ki67, and it was able to differentiate G1 vs. G2 and luminal A vs. luminal B. SUVmax and SUVmean were also significant in both analyses (Table 2 and Table S2). The correlation between SUVmax and Ki67 results was found to be comparable with those described in a previous meta-analysis [45]. Moreover, in a multicenter study, Surov et al. [46] reported a weak correlation between ADCmean and Ki67 in the IDC subgroup, in line with our analysis (see Table S1). Similarly to our work, Incoronato et al. [19] investigated the PET and MRI parameter associations with histopathological factors in IDC. In agreement with our results, they found that Ki67 positive vs. negative and luminal A vs. luminal B are differentiated by SUVmax and SUVmean and not by ADCmean. However, in the same study, they found results opposing to ours regarding tumor grade differentiation. In fact, contrary to our findings, the ADCmean was significant, whereas SUVmax and SUVmean were not significant in the differentiation between low (G1 + G2) vs. high grade (G3). These discrepancies might be related to the different sample size, with their cohort smaller than ours. In another study with a larger population, Jena et al. [47] reported findings similar to ours, observing a significant increase in SUVmax as tumor grade increases.

Regarding the DCE-MRI semi-quantitative parameters, we observed that, in our analysis, none of them are significant. The results on DCE-MRI are controversial in the literature. Some are in line with our findings [48,49,50], while other studies observed the opposite results [51,52,53]. Specifically, Kim et al. [48] found that E_1_ and ESER were not statistically significant in differentiating tumor grade, Ki67, ER, PgR, and molecular subtype, similarly to Fischer et al. [49] and Stomper et al. [50]. However, E_1_ and ESER were found significantly associated with tumor grade in other studies [51,52,53]. In addition, more recently, Niukkanen et al. [54] found intra-tumoral ESER statistically significant in differentiating tumor grade (low vs. high) and Ki67 (negative vs. positive), while intra-tumoral E_1_ did not show any significancy in separating between negative and positive Ki67. These mismatches could be attributed to factors such as the ability to distinguish between intra- and peri-tumoral tissue regions [54], and the inter-observer bias associated with the manual delineation of the VOIs. The DCE-MRI semi-quantitative parameters are descriptors of the signal evolution after the contrast agent injection, and the mean signal in the VOI at each phase depends on the segmented area. The definition of standards for automatic or semi-automatic segmentation techniques may be helpful to reduce variabilities in the results [34].

Among the hybrid parameters, most showed a behavior similar to the [^18^F]FDG PET parameters. This suggests that the correlation with PET components may drive the behavior of hybrid imaging parameters in the analyses. As a matter of fact, almost all hybrid PET/MRI parameters correlate with the percentage value of Ki67 and are significantly able to differentiate tumor grade (G1 vs. G2, G1 vs. G3, Ki67 positive vs. negative, luminal A vs. luminal B) similarly to SUVmax and SUVmean. On the other hand, we observed that SUVmax/Slope and SUVmean/Slope were the only hybrid parameters that are not significant either in the correlations or in the discrimination analysis. Possible explanations may be related to the intrinsic behavior of the Slope parameter [33]. Slope is a descriptor of the post-contrast phase of the TIC. If Slope is lower than −10%, the trend of the TIC is categorized as “wash-out”, while if Slope is between –10% and 10%, the trend of the TIC is classified as “plateau”, otherwise it is defined as “persistent” [33,37,38,39]. Although malignant lesions are generally associated with a Slope lower than 10% [37,38], this is not always the case, since the TIC of the tumor behavior may vary depending on the histotype and even on the acquisition protocol [43]. In fact, in our cohort, we found 62 out of 159 lesions with a “persistent” trend, and this might have influenced the behavior of the corresponding hybrid parameters.

From the ROC analysis, while “single-modality” and “hybrid” parameters have similar AUC and metric values in predicting Ki67 and tumor grade, hybrid parameters could predict luminal A from luminal B better than the single-modality ones. Specifically, SUVmax/ADCmin had higher AUC, sensitivity, specificity, PPV, and NPVs than SUVmax alone. Therefore, hybrid parameters can be used as imaging biomarkers to predict surrogate molecular subtype. This means that the combination of PET and MRI data improves the discrimination of luminal B from luminal A. Similarly to our results, in Catalano et al. [18], a linear discriminant analysis was performed to classify molecular subtypes, and they used SUVmax, ADCmean and Kep_mean_ (mean flux rate constant, a DCE-MRI pharmacokinetic parameter) as input of the prediction model, finding that the PET/MRI data correctly predicted BC phenotype of 14 out of 21 patients compared to biopsy. Previous studies have investigated the importance of molecular subtype discrimination [55,56]. Hung et al. [55] demonstrated that radiotherapy could decrease the likelihood of metastasis in luminal A patients who underwent breast-conserving surgery. Moreover, Mohammed [56] found that patients with luminal A subtypes showed lower mortality rate and higher survival compared to the other molecular subtypes. In conclusion, it can be postulated that the hybrid parameter might help in the formulation of the prognosis in selected cases. However, it is important to emphasize the exploratory nature of this preliminary study. In fact, as shown in Table S1, given the inter-correlation of imaging parameters, a multivariate analysis would be required to support our findings, which are currently based on univariate analyses.

One of the limitations of this study is the class imbalance of tissue biomarkers. Despite the fact that this class imbalance in IDC is also presented in the literature [57], we could not analyze the ability of the imaging biomarker to differentiate between ER- and PgR-positive vs. negative lesions nor perform any analysis on the HER2+ status. Moreover, since there are few samples of HER2+ and triple negative molecular subtypes in the whole dataset, the differentiation analysis on surrogate molecular subtype was evaluated on luminal A and luminal B. A balanced dataset that includes more TN and HER2+ surrogate molecular subtypes would improve the generalizability of the findings. On the other hand, our work is a prospective study, which has the advantage of evaluating a homogeneous cohort acquired with a dedicated hybrid PET/MRI scanner. The second limitation is the use of semi-quantitative DCE-MRI parameters whose results are controversial in the literature, as previously described. Quantitative pharmacokinetic metrics such as K^trans^, Vp, or kep could provide more robust “hybrid parameters”. However, our acquisition protocol, with a temporal resolution of 90 s, is not suitable to calculate these parameters. In our case, this acquisition protocol was chosen to reach high spatial resolution images (incompatible with high temporal resolution) also needed to investigate the axillary lymph nodes. Therefore, a potential development of this work could be the adoption of more recent DCE-MR sequences with both high temporal and spatial resolution [58] to calculate pharmacokinetic parameters which are quantitative descriptors of the blood flow, vessel permeability, and vessel density rather than indirect metrics based on sorely signal intensities.

## 5. Conclusions

This study provides an additional value to hybrid PET/MRI from a quantitative point of view. It was demonstrated that, despite the fact that PET parameters are able to differentiate all the histopathological biomarkers, the combination of PET with MRI parameters demonstrates stronger associations than the two single modalities alone in univariate analysis, specifically, for surrogate molecular subtype prediction. We hypothesize that, in the future, this approach might be helpful in the evaluation of those lesions visible in PET/MRI but not eligible for biopsy, such as metastasis. Therefore, future development include, first, the use of multivariate analyses to confirm our results and, second, the extension of the implementation of this “hybrid parameters” approach to investigate non-biopsy-prone lesions, incorporating different kinds of PET/MRI parameters, such as radiomic parameters.

## Figures and Tables

**Table 1:** Number of lesions for each tissue biomarker. G1: grade 1; G2: grade 2; G3: grade 3; ER: estrogen receptor; PgR: progesterone receptor; Ki67: proliferation index; HER2+: human epidermal growth factor receptor 2 status.

		Number of Lesions (%)
Tumor grade	G1	52 (32.7)
	G2	76 (47.8)
	G3	31 (19.5)
ER	Positive (>0%)	156 (98.1)
	Negative (0%)	3 (1.9)
PgR	Positive (>0%)	157 (98.7)
	Negative (0%)	2 (1.3)
Ki67	Positive (≥20%)	71 (44.7)
	Negative (<20%)	88 (55.3)
HER2+	0	123 (77.4)
	1+	17 (10.7)
	2+	5 (3.1)
	3+	14 (8.8)
Surrogate molecular subtype	Luminal A	87 (54.7)
	Luminal B	52 (32.7)
	Luminal B Her2+	13 (8.2)
	Triple negative	6 (3.8)
	Her2+	1 (0.6)

**Table 2:** List of adjusted *p*-values together with their Hodge-Lehmann Estimator (HLE) and the 99.8% CI of all imaging parameters for each univariate analysis. Significant adjusted *p*-values are in bold.

Parameter		G1 vs. G2	G1 vs. G3	G2 vs. G3	Ki67: Positive vs. Negative	Luminal A vs. Luminal B
E_1_	*p*-value	>0.05	>0.05	>0.05	>0.05	>0.05
	HLE	−10.077	−10.317	−1.867	−7.422	−6.036
	(99.8% CI)	(−46.050–26.400)	(−54.646–35.784)	(−37.282–39.099)	(−37.993–21.57)	(−40.808–26.234)
ESER	*p*-value	>0.05	>0.05	>0.05	>0.05	>0.05
	HLE	−1.860	−4.669	−3.159	−3.100	−3.506
	(99.8% CI)	(−12.290–7.508)	(−16.308–5.589)	(−12.383–6.115)	(−10.705–4.690)	(−12.067–5.495)
Slope	*p*-value	>0.05	>0.05	>0.05	>0.05	>0.05
	HLE	1.865	2.759	0.762	1.047	1.903
	(99.8% CI)	(−6.484–10.626)	(−6.450–11.860)	(−6.751–9.267)	(−5.396–7.846)	(−5.512–9.341)
SPER	*p*-value	>0.05	>0.05	>0.05	>0.05	>0.05
	HLE	−6.759 × 10^−6^	−3.758 × 10^−6^	4.881 × 10^−5^	−3.015 × 10^−5^	−3.015 × 10^−5^
	(99.8% CI)	(−0.039–0.030)	(−0.041–0.040)	(−0.034–0.046)	(−0.024–0.030)	(−0.040–0.029)
SFER	*p*-value	>0.05	>0.05	>0.05	>0.05	>0.05
	HLE	0.040	−0.066	−0.025	−0.033	−0.048
	(99.8% CI)	(−0.207–0.117)	(−0.242–0.122)	(−0.183–0.134)	(−0.162–0.097)	(−0.189–0.106)
ADCmin	*p*-value	**0.0021**	>0.05	>0.05	>0.05	**0.0235**
	HLE	169	145	−23	101	139
	(99.8% CI)	(41–300)	(−56–359)	(−214–159)	(−27–223)	(8–269)
ADCmean	*p*-value	>0.05	>0.05	>0.05	>0.05	>0.05
	HLE	58.127	56.339	−6.661	55.324	79.620
	(99.8% CI)	(−51.673–174.770)	(−105.019–213.593)	(−158.21–146.574)	(−49.950–158.880)	(−17.840–188.465)
SUVmax	*p*-value	**0.0020**	**1.12 × 10^−5^**	**0.0498**	**0.0004**	**0.0068**
	HLE	−2.131	−5.386	−3.410	−2.657	−2.548
	(99.8% CI)	(−4.030–−0.442)	(−10.838–−2.103)	(−8.097–0.017)	(−4.768–−0.745)	(−4.922–−0.368)
SUVmean	*p*-value	**0.0029**	**8.30 × 10^−6^**	**0.0251**	**0.0003**	**0.0065**
	HLE	−1.212	−3.467	−2.290	−1.699	−1.547
	(99.8% CI)	(−2.461–−0.226)	(−6.460–−1.346)	(−4.753–−0.096)	(−2.994–0.460)	(−3.012–−0.199)
SUVmax × E_1_	*p*-value	**0.0115**	**0.0010**	>0.05	**0.0042**	**0.0349**
	HLE	−297.691	−703.845	−411.023	−367.018	−373.674
	(99.8% CI)	(−633.650–−41.390)	(−1606.781–−177.665)	(−1206.068–113.058)	(−761.024–−67.898)	(−831.296–−7.492)
SUVmean × E_1_	*p*-value	**0.0199**	**0.0007**	>0.05	**0.0053**	**0.0491**
	HLE	−172.612	−446.369	−271.128	−223.147	−221.301
	(99.8% CI)	(−382.641–−12.602)	(−925.25–−110.336)	(−722.866–63.257)	(−451.941–−35.899)	(−493.376–1.293)
SUVmax × ESER	*p*-value	**0.0030**	**4.08 × 10^−5^**	>0.05	**0.0008**	**0.0089**
	HLE	−170.235	−442.284	−296.003	−239.514	−240.783
	(99.8% CI)	(−349.988–−31.911)	(−993.297–−145.862)	(−749.033–15.947)	(−418.921–−59.046)	(−455.533–−25.117)
SUVmean × ESER	*p*-value	**0.0064**	**2.31 × 10^−5^**	>0.05	**0.0006**	**0.0096**
	HLE	−101.913	−282.708	−193.411	−151.049	−148.529
	(99.8% CI)	(−206.909–−14.268)	(−586.891–−92.404)	(−431.737–0.891)	(−259.136–−33.863)	(−278.470–−15.998)
SUVmax/Slope	*p*-value	>0.05	>0.05	>0.05	>0.05	>0.05
	HLE	−0.164	−0.702	−0.546	−0.370	−0.541
	(99.8% CI)	(−0.783–0.295)	(−2.269–0.190)	(−2.126–0.766)	(−1.174–0.152)	(−1.682–0.151)
SUVmean/Slope	*p*-value	>0.05	>0.05	>0.05	>0.05	>0.05
	HLE	−0.083	−0.448	−0.371	−0.237	−0.338
	(99.8% CI)	(−0.457–0.191)	(−1.365–0.110)	(−1.266–0.417)	(−0.699–0.083)	(−1.008–0.083)
SUVmax × SPER	*p*-value	**0.0022**	**3.54 × 10^−5^**	>0.05	**0.0007**	**0.0093**
	HLE	−2.283	−5.478	−3.373	−2.766	−2.689
	(99.8% CI)	(−4.400–−0.438)	(−11.810–−1.913)	(−8.805–0.275)	(−5.090–−0.696)	(−5.423–−0.346)
SUVmean × SPER	*p*-value	**0.0039**	**2.00 × 10^−5^**	>0.05	**0.0006**	**0.0098**
	HLE	−1.279	−3.516	−2.362	−1.740	−1.650
	(99.8% CI)	(−2.689–−0.239)	(−7.149–−1.258)	(−5.097–0.056)	(−3.212–−0.433)	(−3.342–−0.180)
SUVmax × SFER	*p*-value	**0.0061**	**0.0001**	>0.05	**0.0021**	**0.0164**
	HLE	−1.726	−4.516	−2.823	−2.354	−2.437
	(99.8% CI)	(−3.680–−0.302)	(−10.729–−1.316)	(−8.249–0.426)	(−4.415–−0.468)	(−4.921–−0.185)
SUVmean × SFER	*p*-value	**0.0126**	**0.0001**	>0.05	**0.0021**	**0.0211**
	HLE	−1.013	−2.859	−1.872	−1.492	−1.503
	(99.8% CI)	(−2.224–−0.088)	(−6.361–−0.865)	(−4.613–0.156)	(−2.689–−0.289)	(−2.957–−0.069)
SUVmax/ADCmin	*p*-value	**3.82 × 10^−5^**	**1.06 × 10^−5^**	>0.05	**0.0001**	**0.0003**
	HLE	−0.005	−0.010	−0.006	−0.005	−0.006
	(99.8% CI)	(−0.009–−0.002)	(−0.023–−0.004)	(−0.016–0.001)	(−0.010–−0.002)	(−0.011–−0.002)
SUVmean/ADCmin	*p*-value	**0.0001**	**1.06 × 10^−5^**	>0.05	**0.0001**	**0.0005**
	HLE	−0.003	−0.006	−0.004	−0.003	−0.003
	(99.8% CI)	(−0.005–−8.542 × 10^−4^)	(−0.014–−0.003)	(−0.010–7.666 × 10^−4^)	(−0.006–−8.825 × 10^−4^)	(−0.007–−8.560 × 10^−4^)
SUVmax/ADCmean	*p*-value	**0.0004**	**1.65 × 10^−5^**	>0.05	**0.0002**	**0.0014**
	HLE	−0.002	−0.005	−0.003	−0.003	−0.003
	(99.8% CI)	(−0.004–−5.992 × 10^−4^)	(−0.012–−0.002)	(−0.009–3.126 × 10^−4^)	(−0.005–−7.600 × 10^−4^)	(−0.005–−5.511 × 10^−4^)
SUVmean/ADCmean	*p*-value	**0.0009**	**1.82 × 10^−5^**	>0.05	**0.0003**	**0.0022**
	HLE	−0.001	−0.003	−0.002	−0.002	−0.002
	(99.8% CI)	(−0.003–−3.168 × 10^−4^)	(−0.008–−0.001)	(−0.006–1.439 × 10^−4^)	(−0.003–−4.762 × 10^−4^)	(−0.003–−3.036 × 10^−4^)

**Table 3:** Comparison between “single-modality” and “hybrid” parameters with the highest AUC score for each group and prediction. Sensitivity, specificity, PPV, and NPV were calculated from the best cutoff chosen by the Youden’s index.

Prediction	Parameter	AUC	Sensitivity (%)	Specificity (%)	PPV (%)	NPV (%)
G1 vs. G2	SUVmax	0.705	84.2	51.9	71.9	69.2
	SUVmax/ADCmin	0.750	64.5	75.0	79.0	59.1
G1 vs. G3	SUVmean	0.836	80.6	78.8	69.4	87.2
	SUVmax/ADCmin	0.833	74.2	82.7	71.9	84.3
G2 vs. G3	SUVmean	0.702	74.2	63.2	45.1	85.7
	SUVmean × ESER	0.689	80.6	51.3	40.3	86.7
Ki67: positive vs. negative	SUVmean	0.703	74.6	58.0	58.9	73.9
	SUVmax/ADCmin	0.710	70.4	63.6	61.0	72.7
Luminal A vs. luminal B	SUVmean	0.685	71.2	60.9	52.1	77.9
	SUVmax/ADCmin	0.720	73.1	63.2	54.3	79.7

## Data Availability

The datasets generated during the current study are available from the corresponding author on reasonable request.

## Supplementary Materials

The following supporting information can be downloaded at: https://www.mdpi.com/article/10.3390/bioengineering13040435/s1, Figure S1: Example of DWI b = 0–1000 s/mm^2^ and the lesion segmentation before and after image registration; Table S1: Correlation among imaging parameters; Table S2: Correlation between imaging parameters and histopathological factors.

## Abbreviations

The following abbreviations are used in this manuscript:

[^18^F]FDG	fluorodeoxyglucose F18
ADC	apparent diffusion coefficient
AUC	area under curve
BC	breast cancer
BIRADS	Breast Imaging Reporting and Data System
CI	confidence interval
CT	computer tomography
DCE	dynamic contrast-enhanced
DWI	diffusion-weighted imaging
E_1_	first post-contrast signal intensity enhancement
ER	estrogen receptor
ESER	early signal enhancement ratio
FOV	field of view
G1	tumor grade 1
G2	tumor grade 2
G3	tumor grade 3
Her2+	human epidermal growth factor receptor 2
IDC	invasive ductal carcinoma
kep	transfer rate from the interstitial to the vascular compartment
Ki67	proliferation index
K^trans^	volumetric transfer constant
MRI	magnetic resonance imaging
MTV	metabolic tumor volume
NPV	negative predictive value
PET	positron emission tomography
PgR	progesterone receptor
PPV	positive predictive value
ROC	receiver operating characteristic
SFER	signal first enhancement ratio
SI	signal intensity
SPER	signal peak enhancement ratio
SUV	standardized uptake value
TB	total body
TE	echo time
TLG	total lesion glycolysis
TOF	time of flight
TR	repetition time
Ve	extravascular extracellular space volume
Vp	plasma volume fraction
VOI	volume of interest
