# Supplementary material for: Hybrid [18F]FDG PET/MR Imaging Parameters for the Prediction of Tissue Biomarkers in Invasive Ductal Breast Cancer
Source: Bioengineering (Basel). 2026 Apr 8;13(4):435. doi: 10.3390/bioengineering13040435 (PMC13113629; doi:10.3390/bioengineering13040435)
Supplement: Supplementary file 1 [file bioengineering-13-00435-s001.zip › Neri et al - Hybrid PETMRI in IDC - Supplementary Material.pdf]

<u>Supplementary Materials of the paper</u>: Hybrid [18F]FDG PET/MR Imaging Parameters for the Prediction of Tissue Biomarkers in Invasive Ductal Breast Cancer

Figure S1: Example of DWI b = 0-1000 s/mm^2 and the lesion segmentation before and after image registration.

Table S1: Correlation among imaging parameters.

Table S2: Correlation between imaging parameters and histopathological factors.

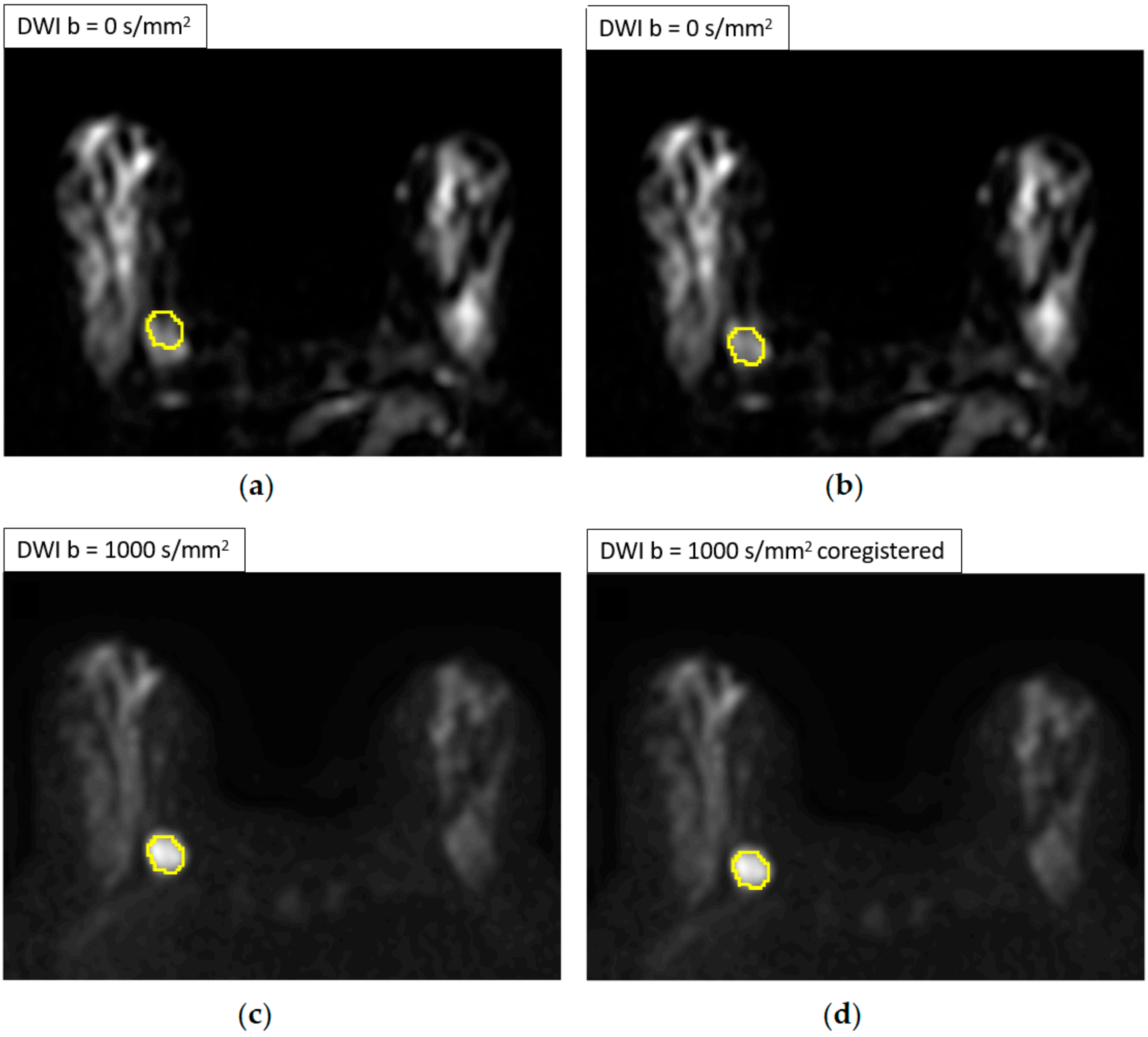

Figure S1: Example of DWI b = 0-1000 s/mm^2 and the lesion segmentation before and after image registration. Example of a 40-years old woman with IDC (grade 2, HER2-negative, luminal B). Lesion segmentation is in yellow. Before registration (**a**, **c**): (**a**) DWI b = 0 s/mm^2; (**c**) DWI b = 1000 s/mm^2. After registration (**b, d**): (**b**) DWI b = 0 s/mm^2 with registered segmentation; (**d**) both DWI b = 1000 s/mm^2 and segmentation registered to DWI b = 0 s/mm^2.

Table S1: Correlation among imaging parameters. Spearman's correlation coefficient matrix between "single modality" (PET and MRI) and "hybrid" (PET/MRI) imaging parameters.

	E_1	ESER	Slope	SPER	SFER	ADCmin	ADCmean	SUVmax	SUVmean
**SUVmax x E_1**	0.6643**	0.6116**	-0.6608**	0.5865**	0.6716**	-0.2609	-0.1578	0.8818**	0.8647**
**SUVmean x E_1**	0.6713**	0.6349**	-0.6798**	0.5946**	0.6906**	-0.2679	-0.1770	0.8552**	0.8899**
**SUVmax x ESER**	0.4129**	0.5907**	-0.5869**	0.4523**	0.6008**	-0.3381*	-0.1983	0.9602**	0.9377**
**SUVmean x ESER**	0.4281**	0.6110**	-0.6078**	0.4635**	0.6207**	-0.3422*	-0.2216	0.9243**	0.9641**
**SUVmax/Slope**	-0.0891	-0.2150	0.2321	-0.2674	-0.2251	-0.0602	0.0150	0.1652	0.1224
**SUVmean/Slope**	-0.0880	-0.2056	0.2284	-0.2705	-0.2195	-0.0540	0.0113	0.1518	0.1241
**SUVmax x SPER**	0.3170*	0.4225**	-0.4631**	0.3943**	0.4591**	-0.3257*	-0.1729	0.9955**	0.9637**
**SUVmean x SPER**	0.3393*	0.4533**	-0.4923**	0.4117**	0.4877**	-0.3362*	-0.2044	0.9626**	0.9957**
**SUVmax x SFER**	0.4844**	0.6474**	-0.6701**	0.5365**	0.6771**	-0.3279*	-0.2029	0.9356**	0.9174**
**SUVmean x SFER**	0.4993**	0.6640**	-0.6869**	0.5458**	0.6928**	-0.3291*	-0.2190	0.9019**	0.9403**
**SUVmax/ADCmin**	0.2241	0.3752*	-0.4033**	0.3251*	0.3988**	-0.6408**	-0.3487*	0.9226**	0.9059**
**SUVmean/ADCmin**	0.2350	0.3901**	-0.4198**	0.3363*	0.4133**	-0.6473**	-0.3675*	0.8973**	0.9257**
**SUVmax/ADCmean**	0.2688	0.3848**	-0.4223**	0.3475*	0.4204**	-0.4434**	-0.3786*	0.9709**	0.9491**
**SUVmean/ADCmean**	0.2836*	0.4115**	-0.4479**	0.3609*	0.4446**	-0.4458**	-0.3996**	0.9340**	0.9749**

*: Significant correlation (0.0001 ≤ adjusted p-value < 0.05)

**: Significant correlation (adjusted p-value < 0.0001)

Table S2: Correlation between imaging parameters and histopathological factors. Spearman's correlation coefficient matrix between imaging parameters and the percentage value of ER, PgR and Ki67.

	ER	PgR	Ki67
**E_1**	-0.1030	0.0220	0.1023
**ESER**	-0.0131	0.1345	0.1573
**Slope**	0.0102	-0.1304	-0.1017
**SPER**	0.0129	0.1059	0.0206
**SFER**	0.0007	0.1452	0.1231
**ADCmin**	-0.0641	0.0891	-0.2833*
**ADCmean**	-0.0469	-0.0183	-0.1820
**SUVmax**	-0.1560	-0.1733	0.4505**
**SUVmean**	-0.1657	-0.1866	0.4607**
**SUVmax x E_1**	-0.1245	-0.1194	0.3848**
**SUVmean x E_1**	-0.1316	-0.1214	0.3889**
**SUVmax x ESER**	-0.1255	-0.1203	0.4323**
**SUVmean x ESER**	-0.1423	-0.1387	0.4378**
**SUVmax/Slope**	-0.0217	-0.1536	0.2060
**SUVmean/Slope**	-0.0231	-0.1546	0.2087
**SUVmax x SPER**	-0.1398	-0.1555	0.4339**
**SUVmean x SPER**	-0.1560	-0.1718	0.4421**
**SUVmax x SFER**	-0.1102	-0.1013	0.4091**
**SUVmean x SFER**	-0.1285	-0.1149	0.4125**
**SUVmax/ADCmin**	-0.1038	-0.1882	0.4847**
**SUVmean/ADCmin**	-0.1063	-0.1943	0.4862**
**SUVmax/ADCmean**	-0.1264	-0.1583	0.4657**
**SUVmean/ADCmean**	-0.1351	-0.1631	0.4660**

*: Significant correlation ($0.0001 \leq$ adjusted p-value < 0.05)

**: Significant correlation (adjusted p-value < 0.0001)
