# Supplementary figures and images for: Hybrid [18F]FDG PET/MR Imaging Parameters for the Prediction of Tissue Biomarkers in Invasive Ductal Breast Cancer
Source: Bioengineering (Basel). 2026 Apr 8;13(4):435. doi: 10.3390/bioengineering13040435 (PMC13113629; doi:10.3390/bioengineering13040435)

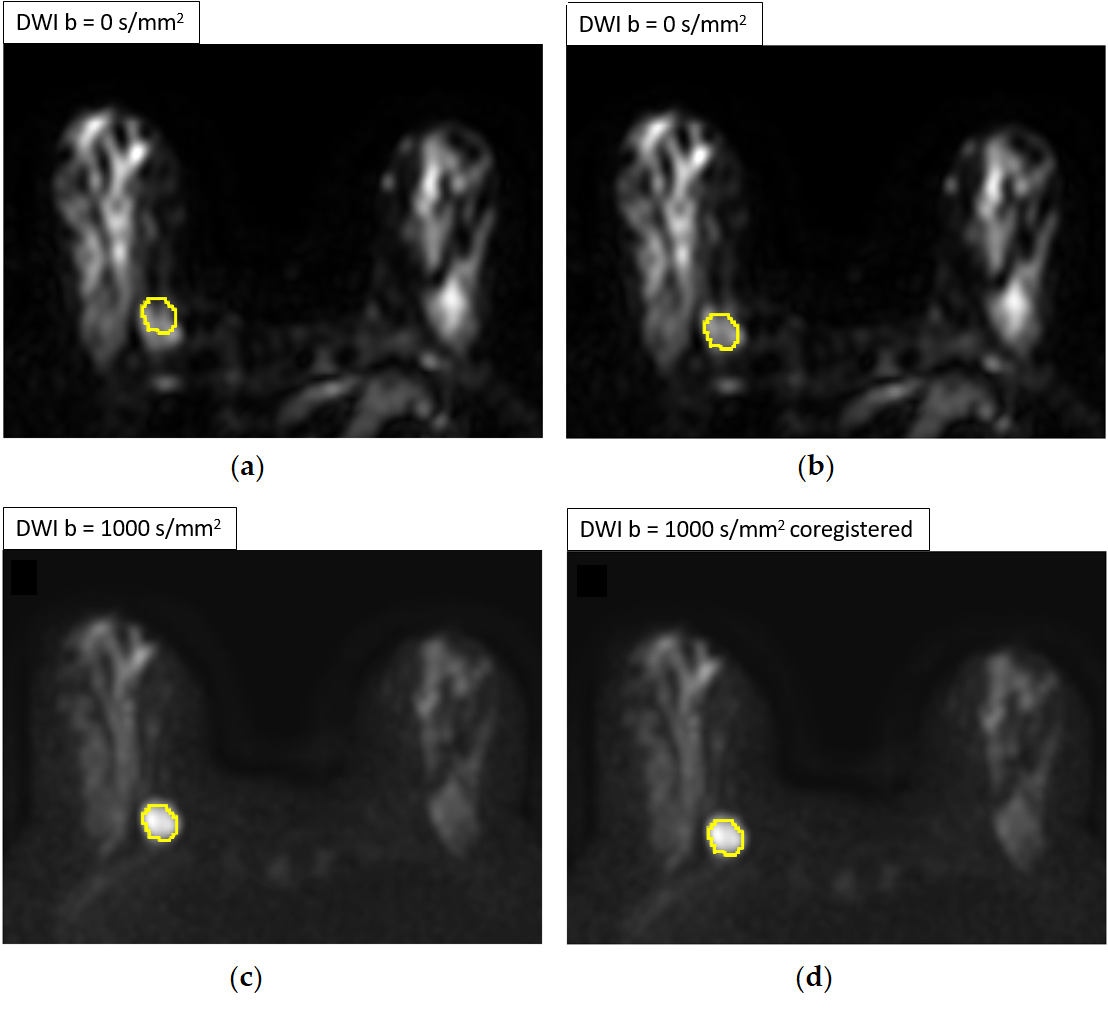

Supplement: Supplementary file 1 [file bioengineering-13-00435-s001.zip › Figure 1 - Supplemental Data.png]
